# The *KLHL40* c.1516A>C is a Chinese‐specific founder mutation causing nemaline myopathy 8: Report of six patients with pre‐ and postnatal phenotypes

**DOI:** 10.1002/mgg3.1229

**Published:** 2020-04-30

**Authors:** Kit San Yeung, Florrie N. Y. Yu, Cheuk Wing Fung, Sheila Wong, Hencher H. C. Lee, Sharon T. H. Fung, Genevieve P. G. Fung, Kwok Yin Leung, Wai Hang Chung, Yun Ting Lee, Vivian K. S. Ng, Mullin H. C. Yu, Jasmine L. F. Fung, Mandy H. Y. Tsang, Kelvin Y. K. Chan, Sophelia H. S. Chan, Anita S. Y. Kan, Brian H. Y. Chung

**Affiliations:** ^1^ Department of Paediatrics and Adolescent Medicine The University of Hong Kong Hong Kong Special Administrative Region Hong Kong China; ^2^ Department of Obstetrics and Gynaecology Queen Elizabeth Hospital Hong Kong Special Administrative Region Hong Kong China; ^3^ Department of Paediatrics and Adolescent Medicine Hong Kong Children's Hospital Hong Kong Special Administrative Region Hong Kong China; ^4^ Department of Pathology Princess Margaret Hospital Hong Kong Special Administrative Region Hong Kong China; ^5^ Department of Paediatrics Kwong Wah Hospital Hong Kong Special Administrative Region Hong Kong China; ^6^ Department of Paediatrics and Adolescent Medicine United Christian Hospital Hong Kong Special Administrative Region Hong Kong China; ^7^ Department of Obstetrics and Gynaecology United Christian Hospital Hong Kong Special Administrative Region Hong Kong China; ^8^ Department of Obstetrics and Gynaecology Princess Margaret Hospital Hong Kong Special Administrative Region Hong Kong China; ^9^ Department of Obstetrics and Gyanecology Kwong Wah Hospital Hong Kong Special Administrative Region Hong Kong China; ^10^ Prenatal Diagnostic Laboratory Department of Obstetrics and Gynaecology Tsan Yuk Hospital Hong Kong Special Administrative Region Hong Kong China; ^11^ Department of Obstetrics and Gynaecology Queen Mary Hospital Hong Kong Special Administrative Region Hong Kong China

**Keywords:** Chinese, founder mutation, *KLHL40*, nemaline myopathy

## Abstract

**Background:**

Autosomal recessive or compound heterozygous mutations in *KLHL40* cause nemaline myopathy 8, which is one of the most severe forms of nemaline myopathy. The *KLHL40* c.1516A>C variant has recently been reported as a founder mutation in southern Chinese.

**Methods:**

We report six cases of nemaline myopathy 8 which involves the c.1516A>C variant, from five unrelated families of non‐consanguineous southern Chinese. The pre‐ and postnatal phenotypes of these cases were reviewed with emphasis on prenatal clinical features. Genetic testing for the founder mutation was performed on three patients with homozygous mutations.

**Results:**

Common prenatal features included reduced fetal movement, polyhydramnios, breech presentation, and clubfeet. Two pregnancies were terminated. Four live‐born patients had postnatal features typical of nemaline myopathy 8. The length of survival ranged from 49 days to 17 months, with respiratory failure and infections being the principal causes of death. Haplotype analysis in three patients with homozygous mutation showed a shared haplotype block of 1.1727 cM spanning over the c.1516A>C variant, suggesting it is a southern Chinese‐specific founder mutation.

**Conclusion:**

Analysis of the *KLHL40* c.1516A>C variant should be considered in prenatal diagnosis of Chinese pregnant patients with suspected congenital neuromuscular disorders or with significant family history of congenital myopathies.

## Graphical abstract

We reported six cases from five unrelated families of non‐consanguineous southern Chinese affected by nemaline myopathy 8, with either homozygous variants or compound heterozygous variants involving c.1516A>C in KLHL40. Pre‐ and postnatal phenotypes of the cases were reviewed, with emphasis on the prenatal clinical features.

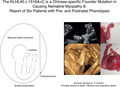

## 1 INTRODUCTION

Nemaline myopathy (NM) is a heterogenous group of congenital myopathies first described by Shy, Engel, Somers, & Wanko (1963). The diagnosis is made by a combination of clinical, histological, and genetic correlations. Clinically, affected individuals present with generalized muscle weakness, hypotonia, depressed deep tendon reflexes, feeding difficulties, respiratory difficulties, and recurrent infections (North & Ryan, 1993). Based on the time of onset and severity of symptoms, it can be classified into six forms ranging from severe congenital/neonatal onset to adult onset (North & Ryan, 1993). Histologically, rod‐like nemaline bodies were observed within muscle nuclei with modified Gömöri trichrome staining (North & Ryan, 1993; Sewry, Laitila, & Wallgren‐Pettersson, 2019). Genetically, mutations in at least 12 genes have been identified to be associated with the condition (Sewry et al., 2019). Incidence of NM appears to be higher in Chinese populations (0.29%) than in other populations (0.02%) (Anderson et al., 2004; Massalska et al., 2016; de Winter & Ottenheijm, 2017; Yin, Pu, Wang, Liu, & Mao, 2014).

Autosomal recessive or compound heterozygous mutations in *KLHL40* (Kelch‐like family member 40, NM_152393.4) cause NM 8 (OMIM #615340), which is one of the severe forms of NM (Ravenscroft et al., 2013). Over 80% of mothers with affected pregnancies present with fetal akinesia/hypokinesia and/or polyhydramnios. Over 90% of affected individuals suffer from respiratory failure in the neonatal period, with the average age of death at 5 months (Ravenscroft et al., 2013). The c.1582G>A p.(Glu528Lys) variant is frequently found in Turkish and Japanese populations suggesting a founder mutation, but a common haplotype has not been identified in either Japanese or Turkish patients (Ravenscroft et al., 2013). The c.1516A>C p.(Thr506Pro) variant in *KLHL40* was first suggested as a hot‐spot mutation in Chinese patients (Chan et al., 2015). Recently, Lee et al. suggested that the c.1516A>C variant is a founder mutation in Chinese patients with NM 8 and hyponatremia (Lee et al., 2019). Here, we present six cases of NM 8 from five unrelated southern Chinese families with no known consanguinity. We reviewed the pre‐ and postnatal phenotypes with special emphasis on the prenatal findings. Three patients (patients 1, 2, and 3) previously reported by Lee et al. (Lee et al., 2019) corresponded to our cases 1, 2, and 4. We used a high‐resolution Chinese‐specific single nucleotide polymorphism (SNP) array to further delineate the genetic distance of the shared haplotype that had a minimum of 1.1727 cM.

## 2 METHODS

### 2.1 Ethical compliance

The study was approved by the institutional review board of the University of Hong Kong/Hospital Authority Hong Kong West Cluster (UW12‐211).

### 2.2 Patients and clinical evaluation

Written informed consent was obtained from the parents of affected individuals. Clinical details were reviewed from the retrieved medical records. DNA samples were obtained from chorionic villi, amniocytes, peripheral blood, or buccal swab. Genetic testing for the founder mutation was carried out using an Infinium OmniZhongHua‐8 v1.4 Kit in three homozygous patients and was performed as previously described (Leung et al., 2017; Yu et al., 2019).

## 3 RESULTS

All unrelated patients carried a homozygous c.1516A>C p.(Thr506Pro) mutation, except for two siblings (cases 2 and 3) who carried compound heterozygous variants c.[1327G>A];[1516A>C] p.[(Gly433Ser)];[(Thr506Pro)]. All the affected individuals inherited the *KLHL40* c.1516A>C variant from one or both of their parents.

### 3.1 Clinical findings (Table 1)

#### 3.1.1 Case 1

A 34‐year‐old nulliparous woman presented with increased serum human chorionic gonadotropin level in the second‐trimester Down syndrome screening, and amniocentesis showed normal karyotype. The anomaly scan was normal. Ultrasound was performed at 36 weeks of gestation for increased symphysial fundal height (42 cm), which showed the fetus in breech presentation with polyhydramnios. She underwent Cesarean section at 36 weeks due to rupture of membranes and 2,700 ml of liquor was noted during delivery. The female baby weighing 2,178 g was born apneic and hypotonic. Multiple abnormalities were noted including increased head circumference, bilateral ptosis, no spontaneous limb movements, multiple joint contractures, and bilateral humeral and femoral fractures. Deltoid muscle biopsy indicated NM. The nerve conduction study showed markedly reduced amplitude due to diminished motor response of atrophic muscles. The baby required ventilation support and tube feeding. The baby passed away at 7 months of age as a result of sudden desaturation and bradycardia.

#### 3.1.2 Cases 2 and 3

A 33‐year‐old nulliparous woman experienced reduced fetal movement. The anomaly scan was normal. The prenatal scan at 32 weeks incidentally detected polyhydramnios. Serial ultrasound showed slowing of fetal growth with progressive polyhydramnios. The scan at 35 weeks showed the fetus was in extended breech presentation with no obvious limb movements. The women underwent Cesarean section at 35 weeks. The male baby weighing 2,210 g was born cyanotic with no respiratory effort. The baby had generalized hypotonia, lack of extraocular muscle and spontaneous limb movements, absent jerks, and bilateral elbow and hip flexion contractures. He required prolonged intubation and tube feeding. The nerve conduction study was normal, and electromyography suggested myopathy. Quadriceps muscle biopsy confirmed NM. The baby had recurrent episodes of aspiration pneumonia and passed away at 16 months of age.

The woman had another spontaneous pregnancy. She underwent chorionic villous sampling which confirmed the presence of the same compound heterozygous variants. Ultrasound examination showed some movement at the fetal knees and forearms, but no obvious movement at the hips or shoulders. Following termination of the pregnancy, thigh muscle biopsy of the abortus did not show typical rod‐like structures in the muscle cells. The electronic microscopy was not performed.

#### 3.1.3 Case 4

A 26‐year‐old nulliparous woman experienced persistently reduced fetal movement. Noninvasive prenatal testing was normal. The anomaly scan showed bilateral clubfeet. Increased head circumference was noted from 26 weeks of gestation. She had spontaneous preterm labor at 31 weeks and the baby was delivered in extended breech presentation vaginally. The female baby weighing 1,440 g was born apneic and flaccid, and had multiple anomalies including disproportionately large head, bilateral ptosis, paralytic hypotonia, abnormal posture with bilateral humeral fracture, lax hip joints, and bilateral clubfeet. Thigh muscle biopsy confirmed NM. Sural nerve biopsy was unrevealing. The baby required high‐flow oxygen ventilation due to carbon dioxide retention, and passed away at 49 days of age due to sepsis.

#### 3.1.4 Case 5

**FIGURE 1:**
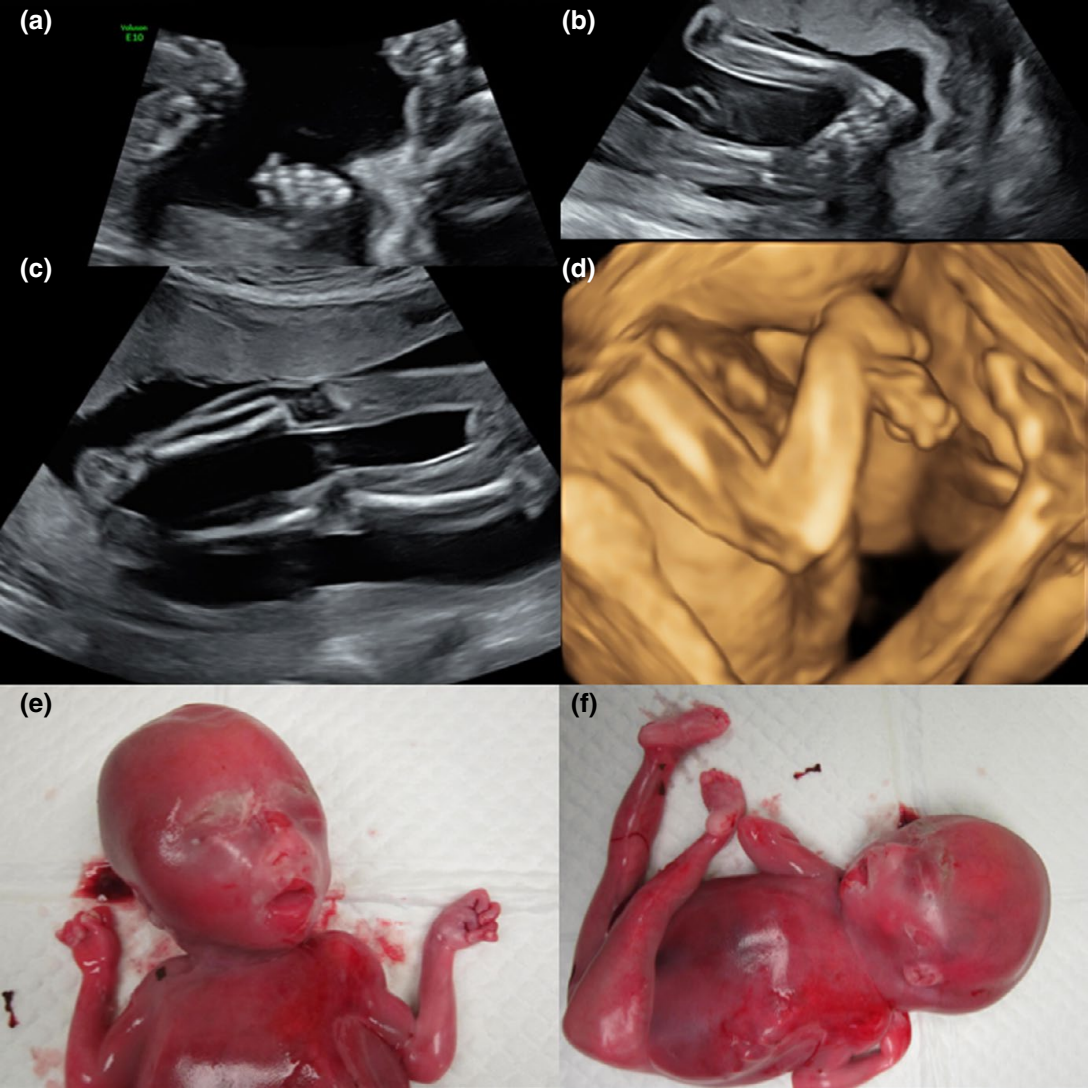
Ultrasound images and clinical photos from case 5. Ultrasound images show closed hand (a), bilateral talipes (b), extended legs (c), and flexed arms and wrists (d). Clinical photos of the abortus show bilateral flexed elbows, closed hands (e), and extended legs with bilateral clubfeet (f)

A 26‐year‐old, gravida 2, and para 1 woman experienced subjectively reduced fetal movement compared with her previous pregnancy. The anomaly scan at 20 weeks of gestation showed a female fetus with abnormal posture, including persistently extended legs, closed hands, flexed wrists, and bilateral clubfeet (Figure 1). Liquor volume was normal. Karyotype and chromosomal microarray of amniocytes were normal. Following termination of the pregnancy, posture of the abortus confirmed the prenatal sonographic findings. Autopsy did not reveal additional structural abnormalities (Figure 1). Muscle biopsy was not performed as a fresh specimen was not available.

#### 3.1.5 Case 6

A 37‐year‐old woman had two unaffected children with another partner. In the index pregnancy, amniocentesis was performed for a positive first‐trimester screening for Down syndrome, which showed normal karyotype. The anomaly scan was normal. She had spontaneous onset of labor at 38 weeks of gestation. The baby was delivered by ventouse extraction, and 4,000 ml amniotic fluid was released upon rupture of membranes. The female baby weighing 2,335 g was born hypotonic with poor respiratory effort. Multiple anomalies were noted including arthrogryposis multiplex congenita, fractured left humerus and right femur, generalized hypotonia, absent spontaneous limb movement, paucity of extraocular muscle movement, and congenital chylothorax. The baby required ventilation support and total parental nutrition. A chest drain was inserted for the chylothorax with a persistently high output. The baby subsequently passed away at 60 days of age.

### 3.2 Founder mutation analysis

**FIGURE 2:**
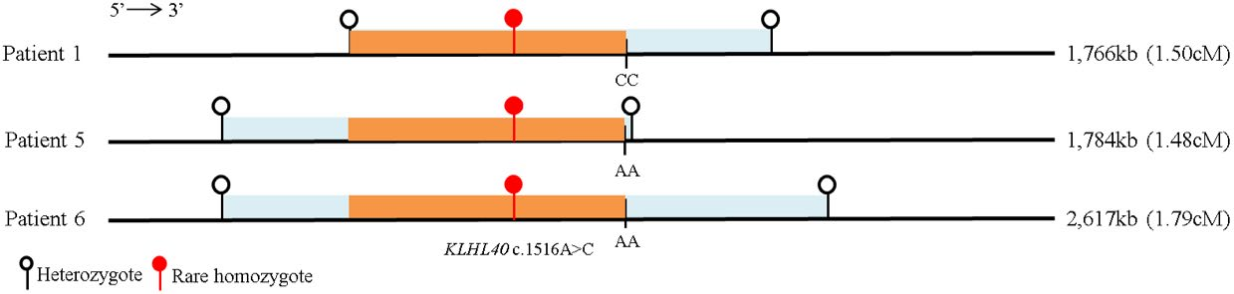
Shared haplotype of homozygous *KLHL40* c.1516A>C. SNP array was used to identify the region of homozygosity across three patients with homozygous c.1516A>C mutation. The closest heterozygous SNP to either side of the c.1516A>C mutation in each patient are indicated by a white circle encompassed by a blue bar. The size of each homozygous segment (i.e., region encompassed by the blue bar) is indicated at the right. Homozygous SNP discrepancy existed in the region of homozygosity among the three patients at the 3′ end (i.e., two patients had an AA genotype and the other had a CC genotype). The length of shared haplotypes was approximately 1,164 kb (highlighted by the orange bar), corresponding to 1.1727 cM

The c.1516A>C variant was reported only in East Asian populations with an allelic frequency of 0.00130 from GnomAD v2.1.1. In our undiagnosed disease exome sequencing cohort collected from Southern Chinese data at the University of Hong Kong (Fung et al., 2019), we found a similar allelic frequency of 0.00138 (3 mutants among 2,174 alleles). Thus, we hypothesized that this is a southern Chinese‐specific founder mutation. Using a high‐resolution Chinese‐specific SNP array, we identified a shared haplotype block of 1,164 kb in length (the genomic coordinate of the shared region is chr3: 42032483–43196801), which corresponded to 1.1727 cM according to HapMap Phase II (Figure 2). This suggests the *KLHL40* c.1516A>C is a Chinese‐specific founder mutation.

## 4 DISCUSSION

Prenatal diagnosis of NM relies on antenatal symptoms, sonographic features, and family history. Prenatally, all our cases had some abnormal clinical or sonographic features, including reduced fetal movement, severe polyhydramnios, clubfeet, arthrogryposis of the hands and feet, and significant family history. The high frequency of prenatal symptoms was consistent with findings by Ravenscroft et al. (2013). However, antenatal diagnosis, which is required for timely counselling of the pregnancy, was established in only two cases (cases 3 and 5). This is probably related to the rarity of the disease and because these features were nonspecific and inconsistent (Massalska et al., 2016). Prenatal detection indicates more severe disease and poorer prognosis (Massalska et al., 2016).

The lack of motility and impaired swallowing result in polyhydramnios (Vardon, Chau, Sigodi, Figarella‐Branger, & Boubli, 1998). In our three live‐born patients with polyhydramnios, these features were evident at 32 weeks (case 2), 36 weeks (case 1), and 38 weeks (case 6). Abnormal muscle development and fiber differentiation need to reach a critical stage to cause significant changes in fetal motility, which may have late onset (Mulder, Nikkels, & Visser, 2001). Although reduction in fetal movement may only be present in the third trimester or even retrospectively, it can be observed on the prenatal scan (as in cases 2, 3, and 5). Assessment of fetal movement is time‐consuming and may require serial scans (Donker, Eijckelhof, Tan, & Vries, 2009). Polyhydramnios and absence of fetal movement on ultrasound appear to be associated with increased risk of mortality (Vardon et al., 1998).

All our live‐born patients had ophthalmoparesis and pathological fractures, which are relatively common in *KLHL40* mutations (Ravenscroft et al., 2013). Three out of four affected babies were delivered in breech presentation, which could be attributed to the relatively large head size, fetal hypokinesia, persistent leg extension, and polyhydramnios. Survival of our live‐born patients was on average 6 months and ranged from 49 days to 17 months, which is compatible with the findings from Ravenscroft et al. (2013). Respiratory failure and infections were the major causes of death.

## 5 CONCLUSION

The analysis of six cases of NM 8 showed the c.1516A>C variant in *KLHL40* is a Chinese‐specific founder mutation. We suggest that *KLHL40* c.1516A>C variant should be considered in prenatal diagnosis of Chinese pregnant patients with suspected congenital neuromuscular disorders or with significant family history of congenital myopathies.

## DISCLOSURE OF INTERESTS

The authors have no conflict of interest to disclose.

## AUTHORS CONTRIBUTION

Kit‐san Yeung and Florrie NY Yu drafted the manuscript. Sophelia HS Chan, Anita SY Kan, and Brian HY Chung designed the project, supervised and revised the intellectual content of the manuscript. Florrie NY Yu, Kwok Yin Leung, Vivian KS Ng, Wai Hung Chung, and Yun Ting Lee provided the prenatal clinical data. Cheuk Wing Fung, Shelia Wong, Hencher Lee, and Sharon TH Fung and Genevieve PG Fung provided the postnatal clinical data. Kit‐san Yeung, Mullin HC Yu, Jasmine LF Fung, Mandy HY Tsang, and Kelvin YK Chan performed the genetic analysis.

6

**TABLE 1:** Antenatal features, ultrasound features, clinical features, muscle biopsy findings, and outcomes in six cases of nemaline myopathy

Case	Gestation at delivery (weeks)	Gestation at TOP (weeks)	Antenatal features	Ultrasound features	Postnatal features	Mode of delivery	Birth weight (g)	Muscle biopsy findings	Source of genetic testing	Outcome
1	36	N/A	Large‐for‐date uterus	Polyhydramnios, breech presentation	Hypotonia, increased head circumference, bilateral ptosis, lack of facial expression, multiple limb joint contractures, no spontaneous limb movements, bilateral humeral and femoral fractures and sialorrhea	Cesarean section	2,178	Nemaline myopathy	Peripheral blood	Live birth, death at 7 months of age
2	35	N/A	Reduced fetal movement	Fetal growth restriction, extended breech presentation, reduced limb movements, polyhydramnios	Generalized hypotonia, tented mouth, frog‐like posture, lack of facial and extraocular muscle movement, lack of spontaneous limb movement, absent jerks, wasting over bilateral quadriceps, bilateral elbow and hip flexion contractures	Cesarean section	2,210	Nemaline myopathy	Peripheral blood	Live birth, death at 17 months of age
3	N/A	14	Previously affected pregnancy	No obvious movement at hip or shoulders	Not available	N/A	N/A	Typical rod‐like bodies not identified	Chorionic villi	TOP
4	31	N/A	Reduced fetal movement	Bilateral clubfeet, increased head circumference, extended breech presentation	Paralytic hypotonia, downslanting palpebral fissures, large head with frontal bossing, bilateral ptosis, lack of facial movements, lax hip, bilateral clubfeet, bilateral humeral fracture	Assisted vaginal breech delivery	1,440	Nemaline myopathy	Peripheral blood	Live birth, death at 49 days of age
5	N/A	22	Reduced fetal movement	Persistently extended legs, bilateral clubfeet, flexed wrists, closed hands	Not available	N/A	N/A	Not done	Amniocytes	TOP
6	38	N/A	Polyhydramnios	Normal anomaly scan	Generalized hypotonia, hypoventilation, receding chin, arthrogryposis multiplex congenita, absent gag reflex, absent spontaneous limb movements, paucity of extraocular muscle movements, congenital chylothorax, fractured left humerus, and right femur	Cesarean section	2,335	Not done	Buccal swab	Live birth, death at 60 days of age

Abbreviations: N/A, Information not applicable; TOP, Termination of pregnancy.
